# Association of age with response to preoperative chemotherapy in patients with muscle-invasive bladder cancer

**DOI:** 10.1007/s00345-021-03793-4

**Published:** 2021-08-09

**Authors:** David D’Andrea, Peter C. Black, Homayoun Zargar, Kamran Zargar-Shoshtari, Francesco Soria, Adrian S. Fairey, Laura S. Mertens, Colin P. Dinney, Maria C. Mir, Laura-Maria Krabbe, Michael S. Cookson, Niels-Erik Jacobsen, Jeffrey S. Montgomery, Nikhil Vasdev, Evan Y. Yu, Evanguelos Xylinas, Nicholas J. Campain, Wassim Kassouf, Marc A. Dall’Era, Jo-An Seah, Cesar E. Ercole, Simon Horenblas, Srikala S. Sridhar, John S. McGrath, Jonathan Aning, Jonathan L. Wright, Andrew C. Thorpe, Todd M. Morgan, Jeff M. Holzbeierlein, Trinity J. Bivalacqua, Scott North, Daniel A. Barocas, Yair Lotan, Petros Grivas, Andrew J. Stephenson, Jay B. Shah, Bas W. van Rhijn, Siamak Daneshmand, Philippe E. Spiess, Shahrokh F. Shariat

**Affiliations:** 1 Department of Urology, Comprehensive Cancer Center, Medical University of Vienna, Währinger Gürtel 18-20, 1090 Vienna, Austria; 2 Department of Urologic Sciences, University of British Columbia, Vancouver, BC Canada; 3 Department of Urology, Western Health, Melbourne, Australia; 4 Department of Genitourinary Oncology, H Lee Moffitt Cancer Center and Research Institute, Tampa, FL USA; 5 University of Auckland, Auckland, New Zealand; 6 Department of Urology, Molinette Hospital, University of Turin, Turin, Italy; 7 University of Alberta, Edmonton, AB Canada; 8 Department of Urology, The Netherlands Cancer Institute - Antoni Van Leeuwenhoek Hospital, Amsterdam, The Netherlands; 9 Department of Urology, MD Anderson Cancer Center, Houston, TX USA; 10 Glickman Urological and Kidney Institute, Cleveland Clinic, Cleveland, OH USA; 11 Department of Urology, Fundacion Instituto Valenciano de Oncologia, Valencia, Spain; 12 Department of Urology, University of Texas Southwestern Medical Center, Dallas, TX USA; 13 Department of Urology, University of Münster, Münster, Germany; 14 Department of Urology, University of Oklahoma College of Medicine, Oklahoma City, OK USA; 15 Department of Urology, University of Michigan Health System, Ann Arbor, MI USA; 16 Hertfordshire and Bedfordshire Urological Cancer Centre, Department of Urology, Lister Hospital, Stevenage, UK; 17 Department of Urology, Freeman Hospital, Newcastle Upon Tyne, UK; 18 Department of Medicine, Division of Medical Oncology, University of Washington School of Medicine and Fred Hutchinson Cancer Research Center, Seattle, WA USA; 19 Department of Urology, Hôpital Bichat-Claude Bernard, Assistance Publique-Hôpitaux de Paris, Université de Paris, Paris, France; 20 Department of Surgery, Exeter Surgical Health Services Research Unit, Royal Devon and Exeter NHS Trust, Exeter, UK; 21 Department of Surgery (Division of Urology), McGill University Health Center, Montreal, Canada; 22 Department of Urology, Davis Medical Center, University of California At Davis, Sacramento, CA USA; 23 Princess Margaret Hospital, Toronto, ON Canada; 24 Bristol Urological Institute, North Bristol NHS Trust, Bristol, UK; 25 Department of Urology, University of Washington, Seattle, WA USA; 26 Department of Urology, University of Kansas Medical Center, Kansas City, KS USA; 27 Department of Urology, The James Buchanan Brady Urological Institute, The Johns Hopkins School of Medicine, Baltimore, MD USA; 28 Cross Cancer Institute, Edmonton, AB Canada; 29 Department of Oncology, University of Alberta, Edmonton, AB Canada; 30 Department of Urologic Surgery, Vanderbilt University Medical Center, Nashville, TN USA; 31 Department of Hematology and Medical Oncology, Taussig Cancer Institute, Cleveland Clinic, Cleveland, USA; 32 Department of Urology, Stanford University School of Medicine, Stanford, CA USA; 33 USC/Norris Comprehensive Cancer Center, Institute of Urology, University of Southern California, Los Angeles, CA USA; 34 Departments of Urology, Weill Cornell Medical College, New York, NY USA; 35 Department of Urology, Second Faculty of Medicine, Charles University, Prag, Czech Republic; 36 Institute for Urology and Reproductive Health, I.M. Sechenov First Moscow State Medical University, Moscow, Russia

**Keywords:** Bladder cancer, Chemotherapy, Age, Response

## Abstract

**Purpose:**

To assess the association of patient age with response to preoperative chemotherapy in patients with muscle-invasive bladder cancer (MIBC).

**Materials and methods:**

We analyzed data from 1105 patients with MIBC. Patients age was evaluated as continuous variable and stratified in quartiles. Pathologic objective response (pOR; ypT0-Ta-Tis-T1N0) and pathologic complete response (pCR; ypT0N0), as well survival outcomes were assessed. We used data of 395 patients from The Cancer Genome Atlas (TCGA) to investigate the prevalence of TCGA molecular subtypes and DNA damage repair (DDR) gene alterations according to patient age.

**Results:**

pOR was achieved in 40% of patients. There was no difference in distribution of pOR or pCR between age quartiles. On univariable logistic regression analysis, patient age was not associated with pOR or pCR when evaluated as continuous variables or stratified in quartiles (all *p* > 0.3). Median follow-up was 18 months (IQR 6–37). On Cox regression and competing risk regression analyses, age was not associated with survival outcomes (all *p* > 0.05). In the TCGA cohort, patient with age ≤ 60 years has 7% less DDR gene mutations (*p* = 0.59). We found higher age distribution in patients with luminal (*p* < 0.001) and luminal infiltrated (*p* = 0.002) compared to those with luminal papillary subtype.

**Conclusions:**

While younger patients may have less mutational tumor burden, our analysis failed to show an association of age with response to preoperative chemotherapy or survival outcomes. Therefore, the use of preoperative chemotherapy should be considered regardless of patient age.

**Supplementary Information:**

The online version contains supplementary material available at 10.1007/s00345-021-03793-4.

## Introduction

Neoadjuvant chemotherapy (NAC) followed by radical cystectomy (RC) and pelvic lymphadenectomy is the standard of care for muscle invasive bladder cancer (MIBC) [1].

While NAC has shown to improve survival [2–4], not every patient will respond to this preoperative therapy [5].

Identification of patients who are unlikely to respond to NAC is of paramount importance for clinical decision making and patient counseling to avoid overtreatment and minimize unnecessary adverse events. This is specifically true for bladder cancer (BC) patients as they have, in general, various comorbidities and are often frail [6, 7]. While several factors, such as clinical tumor stage, histological variants, patient sex, exposure to carcinogens and tumor mutational burden haven been linked to the response to NAC [5, 8–15], only little is known about the association of age with response to and survival after NAC and RC. A recent analysis of The Cancer Genome Atlas (TCGA) showed an age-related distribution of total mutational burden, neoantigen load, molecular subtypes and intra-tumoral immune signaling in MIBC [16]. Based on these findings, we hypothesize that there might be an age-dependent response to preoperative chemotherapy.

To address this question, we analyzed the data originating from a multicenter cooperation on preoperative chemotherapy in RC.

## Materials and methods

### Patient selection and intervention

We retrospectively reviewed our multicenter database of 1543 patients treated with preoperative chemotherapy followed by RC and lymphadenectomy between 2000 and 2013.

Patients who received less than 2 cycles of preoperative chemotherapy (*n* = 151), those with unknown clinical stage (*n* = 24), those with unknown pathological stage (*n* = 53) and those who did not receive cisplatin-based combination chemotherapy (*n* = 210) were removed, leaving 1105 patients for final analyses (supplementary figure S1). No patient had clinically distant metastases on preoperative imaging.

Preoperative chemotherapy regimens consisted of methotrexate, vinblastine, doxorubicin, and cisplatin (MVAC), dose dense MVAC (ddMVAC) or gemcitabine and cisplatin (GemCis). The chemotherapy regimen and number of cycles were administered at clinician discretion and according to institutional standards.

All RC procedures were performed by an open technique. The decision for the type of urinary diversion was based on disease characteristics, patient wishes and performance status. All surgical specimens were processed according to standard pathologic procedures and staged according to the TNM classification.

### Outcome measurement

Primary outcome of the study was pathologic objective response (pOR), defined as ypT0-Ta-Tis-T1N0. Secondary outcomes of the study were pathologic complete response (pCR), defined as ypT0N0, overall survival (OS) and cancer-specific survival (CSS).

Time-to-event was calculated from the administration of the first chemotherapy cycle until the last follow-up. Cause of death was recorded through patient chart review or death certificates [17]. We evaluated age as continuous variable and stratified the population based on age quartiles.

### Age and molecular landscape

We used data from 395 TCGA patients [18] to investigate the prevalence of TCGA molecular subtypes (luminal papillary, luminal infiltrated, luminal, basal squamous and neuronal) and DNA damage repair (DDR) gene alterations according to patient age. We selected ERCC2, RB1, ATM, FANCC ATR, BRCA1, BRCA2, ERCC5, RAD51C, and REQLC4 as key DDR genes based on prior reports and current ongoing prospective trials [14, 19, 20].

### Statistical analyses

We compared the distribution of clinicopathologic features between age groups using the chi-square test for categorical variables and the Mann–Whitney-U test for continuous variables. We evaluated the association of patient age with pathologic response using univariable and multivariable logistic regression modeling. Due to the even distribution of the data between groups, adjustments using, i.e., propensity score were not applied.

We used two different approaches for the time-to-event analysis. First, we used the Cox regression analysis to investigate the association of age with OS and CSS. Survival functions were plotted using the Kaplan–Maier estimates. Second, we estimated the marginal probability of death from BC using competing risk analysis where death of other cause was considered the competing event. The proportional hazard was modeled using the Fine and Gray function.

We investigated the validity of the survival model testing the proportional hazard assumption and visually assessed the functional form of the association of age with cancer-specific death using the plot of Martingale residuals from a null Cox model against age.

Due to the exploratory character of the study, statistical significance was considered at *p* < 0.05, but not in a confirmatory manner. Therefore, no adjustment for multiplicity was performed. Statistical analyses were performed with R (The R Project, Vienna, Austria).

## Results

Overall, 437 (40%) patients had a pOR and 234 (21%) had pCR. There was no difference in clinicopathologic features, distribution in pOR or pCR between age quartiles (Table 1).

**Table 1:** Clinicopathologic features of 1,105 patients treated with neoadjuvant chemotherapy followed by radical cystectomy and lymphadenectomy for muscle-invasive bladder cancer, stratified by age quartiles

Variable	Overall, *N* = 1105^a^	27-57, *N* = 280^a^	58-64, *N* = 288^a^	65-71, *N* = 290^a^	72-87, *N* = 247^a^	*p*-value^b^
Sex						> 0.9
Female	258 (23%)	63 (22%)	70 (24%)	70 (24%)	55 (22%)	
Male	847 (77%)	217 (78%)	218 (76%)	220 (76%)	192 (78%)	
cT stage						> 0.9
cT2	680 (62%)	170 (61%)	178 (62%)	180 (62%)	152 (62%)	
cT3	299 (27%)	75 (27%)	78 (27%)	80 (28%)	66 (27%)	
cT4	126 (11%)	35 (12%)	32 (11%)	30 (10%)	29 (12%)	
NAC regimen						0.11
ddMVAC	139 (13%)	35 (12%)	45 (16%)	36 (12%)	23 (9.3%)	
GEM–CIS	813 (74%)	195 (70%)	206 (72%)	220 (76%)	192 (78%)	
MVAC	153 (14%)	50 (18%)	37 (13%)	34 (12%)	32 (13%)	
NAC cycles						0.11
2–4	1,028 (93%)	267 (95%)	260 (90%)	272 (94%)	229 (93%)	
5 or more	77 (7.0%)	13 (4.6%)	28 (9.7%)	18 (6.2%)	18 (7.3%)	
ypT stage						0.6
ypT0	251 (23%)	67 (24%)	63 (22%)	62 (21%)	59 (24%)	
ypT1-Ta-Tis	226 (20%)	49 (18%)	72 (25%)	61 (21%)	44 (18%)	
ypT2	203 (18%)	53 (19%)	45 (16%)	57 (20%)	48 (19%)	
ypT3–T4	425 (38%)	111 (40%)	108 (38%)	110 (38%)	96 (39%)	
ypN stage						0.9
ypN0	818 (74%)	213 (76%)	213 (74%)	212 (73%)	180 (73%)	
ypN1	103 (9.3%)	24 (8.6%)	26 (9.0%)	27 (9.3%)	26 (11%)	
ypN2	153 (14%)	37 (13%)	37 (13%)	43 (15%)	36 (15%)	
ypN3	31 (2.8%)	6 (2.1%)	12 (4.2%)	8 (2.8%)	5 (2.0%)	
Variant histology	99 (9.0%)	25 (8.9%)	21 (7.3%)	28 (9.7%)	25 (10%)	0.7
Lymph nodes removed	19 (12, 28)	19 (13, 28)	18 (11, 28)	19 (13, 28)	19 (12, 27)	0.6
STSM						> 0.9
Negative	918 (83%)	233 (83%)	240 (83%)	241 (83%)	204 (83%)	
Positive	81 (7.3%)	21 (7.5%)	22 (7.6%)	20 (6.9%)	18 (7.3%)	
Not evaluable	106 (9.6%)	26 (9.3%)	26 (9.0%)	29 (10%)	25 (10%)	
pOR	437 (40%)	112 (40%)	124 (43%)	110 (38%)	91 (37%)	0.5
pCR	234 (21%)	65 (23%)	60 (21%)	57 (20%)	52 (21%)	0.8

MVAC: methotrexate, vinblastine, doxorubicin, cisplatin; ddMVAC: dose dense methotrexate, vinblastine, doxorubicin, cisplatin; GEM–CIS: gemcitabine–cisplatin; STSM: soft tissue surgical margin; pOR: pathologic objective response; pCR: pathologic complete response

^a^Median (IQR) or Frequency (%)

^b^Pearson's Chi-squared test; Kruskal–Wallis rank sum test

For the primary endpoint, on univariable logistic regression analysis, patient age was not associated with pOR (OR 1.00; 95% CI 0.99–1.01; *p* = 0.7), pCR (OR 1.00; 95% CI 0.99–1.02; *p* = 0.9) when evaluated as continuous variable. When the cohort was divided in quartiles, there was no association of patients age with outcomes (all *p* > 0.5, supplementary Table S1).

Overall, 139 patients had insufficient follow-up, leaving 966 patients for survival analyses.

**Fig. 1:**
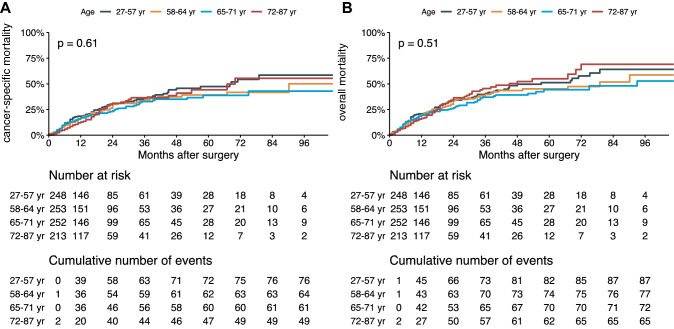
Survival function estimating the cancer-specific mortality (**A**) and overall mortality (**B**) in 966 patients treated with preoperative chemotherapy and radical cystectomy for muscle-invasive bladder cancer, stratified by age

During a median follow-up for alive patients of 18 months (IQR 6–37), 303 (31%) died of any cause and 250 (21%) died of BC. On Cox regression analysis, age evaluated as continuous variable or stratified in quartiles was not associated with CSS or OS (all *p* > 0.2). Similarly, on competing risk regression analysis age was not associated with cancer-specific death (all *p* > 0.052) (Fig. 1 and Table 2). The proportional hazard assumption was not violated (*p* = 0.72), confirming the validity of the model (Supplementary Figure S2a). The Martingale residuals plot did not show an association of age with cancer-specific death (Supplementary Figure S2b).

**Table 2:** Survival analyses investigating the association of age with cancer-specific survival (CSS), overall survival (OS) and death from bladder cancer in 966 patients treated with preoperative chemotherapy followed by radical cystectomy and lymphadenectomy

	Overall survival			Cancer-specific survival			Fine and Gray model		
	HR	95% CI	*p*-value	HR	95% CI	*p*-value	HR	95% CI	*p*-value
Age (years)	1.00	0.98, 1.01	0.6	0.99	0.98, 1.00	0.11	0.99	0.97–1.00	0.052
Age groups									
27–57	–	–		–	–			–	–
58–64	0.89	0.66, 1.21	0.5	0.85	0.61, 1.19	0.3	0.83	0.59–1.16	0.27
65–71	0.82	0.60, 1.11	0.2	0.80	0.57, 1.12	0.2	0.80	0.58–1.12	0.20
72–87	1.01	0.74, 1.40	> 0.9	0.88	0.62, 1.26	0.5	0.81	0.57–1.15	0.25

HR: Hazard Ratio; CI: Confidence Interval

In a subgroup analysis of patients with pOR, age evaluated as continuous variable or stratified in quartiles was not associated with CSS, OS or cancer-specific death (Supplementary table S2).

**Fig. 2:**
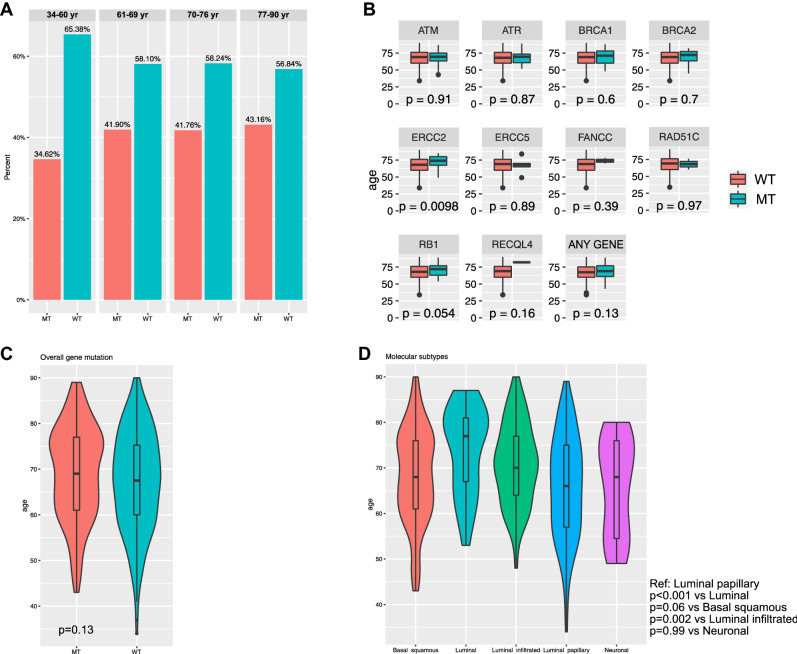
**A** Prevalence of selected DNA damage repair (DDR) gene mutations in 395 patients with muscle-invasive bladder cancer stratified by age quartiles. **B** Distribution of selected DDR gene mutations according to age. **C** Distribution of selected DDR gene mutations according to age. **D** Distribution of mRNA cluster molecular subtypes according to age. Data extracted from The Cancer Genome Atlas [18]. WT: wild type; MT: mutated

Analyzing the TCGA cohort, we found that 35%, 42%, 42% and 43% of patients aged 34–60, 61–69, 70–76 and 77–90 years had at least one mutation in the selected DDR gene panel, respectively (Fig. 2A, *p* = 0.59). When age was analyzed as a continuous variable, we found an association of ERCC2 mutations with older age (Fig. 2B). There was no difference in the distribution of age according to overall gene mutation (Fig. 2C, *p* = 0.13) Finally, we found older age distribution in patients with luminal (*p* < 0.001) and luminal infiltrated (*p* = 0.002) compared to those with luminal papillary molecular subtype (Fig. 2D).

## Discussion

We investigated the association of patient age with pathologic response and survival after preoperative chemotherapy using a large multicenter cohort and found no association of age evaluated as continuous variable or stratified in quartiles. Moreover, we found no association of age with CSS, OS or cancer-specific death.

The risk of BC incidence increases with age [21]. This is attributed to several factors including cumulative exposure to carcinogens and increasing genetic mutations [22–24]. It has been postulated that the evolving mutational burden of MIBC could have an influence on response to systemic therapy [16]. While several genetic and pathologic features have been associated with response to preoperative chemotherapy [12, 14, 25–27], current literature shows controversial results regarding the role of age [28]. The evidence provided by large population-based cancer registry studies is often missing analyses investigating the association of age with therapy response and survival in patients treated with preoperative chemotherapy and RC [29, 30], and meta-analyses of randomized controlled trials showed that only the minority of patients treated with RC and preoperative chemotherapy are > 65 years [2, 3].

The randomized SWOG-8710 trial compared the effect of NAC plus RC with RC alone in patients with MIBC [4]. The investigators performed subgroup analyses based on patient age using the median age of 64 years as the cut-off. They reported a median OS in patients ≤ 64 years of 104 months compared to 61 months in patients > 64 years (*p* = 0.05). In SWOG-8710, the association of pathologic response rates with age was not investigated. Pathologic response to chemotherapy is a generally accepted surrogate marker for survival in a trial level and could be influenced by patient age. In an analysis of 189 patients originating from the Retrospective International Study of Cancers of the Urothelial Tract (RISC), authors found an association between pathologic downstaging and response to chemotherapy with improved survival in MIBC. Authors performed an external validation of these finds in 2010 patients originating from the National Cancer Database (NCDB) [31]. In this report, age was included as continuous variable in the multivariable analysis and was significantly associated with outcomes in the NCDB cohort but not in the RISC cohort. In a multicenter retrospective study of 640 patients, authors aimed to identify prognostic features for cancer-specific death after NAC. While age was significantly associated with cancer-specific death on univariable Cox analysis, this association disappeared in the multivariable analysis and was, therefore, not included in the prognostic model [32]. Similarly, single-center series reported no difference in pathologic response or survival between younger and older patients [33, 34].

When interpreting these results, one could assume that the statistical non-significant association of age with outcomes is because retrospective studies are underpowered and prospective trials are not powered for the secondary endpoints. This can partially explain our findings that failed to show an association of age with oncologic outcomes. Indeed, the number of patients included in our study was probably too small to detect a significant difference between cohorts. Corroboration of these finding in a larger cohort could shed light on this.

Moreover, it should also be considered that the association of age with oncologic outcomes in MIBC is not only dependent on the biology of the disease itself, but also on barriers to access health care and sub-optimal treatment in older patients. Indeed, these patients may tend to receive less aggressive surgeries and sub-therapeutic dosing of systemic therapies [28, 29, 35–39]. Moreover, it has been shown that patients included in clinical trials only marginally reflect the real-world scenario. Indeed, only 15% of clinical trials could feasibly be replicated using currently available real-world data sources [40]. With this study, we complement the literature to help inform clinical practice, regarding patient selection and counseling.

The mutational landscape of UCB has been a particular focus of recent research. An analysis of the TGCA project has shown an association of somatic mutation rate with age in patients with MIBC [16]. This generates the hypothesis that there might be an age-dependent response to preoperative chemotherapy. We corroborated these findings with a granular analysis of the TCGA project investigating a panel of established DRR genes and molecular subtypes and their association with age. Younger patients had less DDR gene mutations while older patients had more luminal and luminal infiltrated molecular subtypes. Moreover, we found a significantly different distribution of age in patients with ERCC2 mutation. Molecular subtypes have been associated with response to preoperative chemotherapy and survival. Specifically, luminal infiltrated tumors showed lower response rates and survival while luminal papillary showed better survival rates, regardless of preoperative chemotherapy [25]. However, these findings need further exploration in well-designed prospective clinical trials.

Despite all its strengths, our study is not devoid of limitations which are mainly inherent in its retrospective design. We had no information on smoking status, exposure to chemical compounds and renal function. Therefore, we could not adjust our analyses for the effect of these variables. We could not adjust for the effects of complications that occurred during preoperative chemotherapy resulting in subsequent dose reduction or sub-therapeutic dosing. There was no central pathological review of the specimens. The preoperative staging was not standardized and we had no information on preoperative comorbidities assessed by a validated score. Moreover, variability in standard practices, quality of surgery, frequency of surveillance imaging, sarcopenia and nutritional status in the elderly [41], as well as other selection biases and other not measurable confounders, may have influenced our results.

Development and identification of predictive and prognostic tools based on clinical variables and molecular biomarkers is essential for accurate identification of patients who are more likely to benefit from preoperative chemotherapy. In our analysis, we failed to prove an association of age with response to preoperative chemotherapy and survival in patients treated with RC. Our findings support the administration of preoperative chemotherapy, in patients who can tolerate it, regardless of their age. This hypothesis should be further investigated in prospective clinical trials and large, comparative retrospective cohorts.

## Conclusion

This study found that age is not associated with response to preoperative chemotherapy. Therefore, it should be offered to patients regardless of age but rather based on their overall performance status and underlying renal function. While the TCGA analysis showed that younger patients may have less mutational tumor burden, this factor may not translate into response to preoperative chemotherapy and further research is needing into the impact of genetic factors and response to systemic therapy.

## Supplementary Information

Below is the link to the electronic supplementary material.

Supplementary file1 (DOCX 261 KB)

## Data Availability

Data can be provided on request.

## Code availability

Programming codes can be provided on request.
