# Supplementary material for: Association of age with response to preoperative chemotherapy in patients with muscle-invasive bladder cancer
Source: World J Urol. 2021 Aug 9;39(12):4345–54. doi: 10.1007/s00345-021-03793-4 (PMC8602146; doi:10.1007/s00345-021-03793-4)
Supplement: Supplementary file 1 — Supplementary file1 (DOCX 261 KB) [file 345_2021_3793_MOESM1_ESM.docx]

**Supplementary Figure S1** Patient selection process

**Supplementary Figure S2** (A) Plot of age against Martingale residuals and Schoenfeld residuals (B) from a null Cox model for cancer-specific death in 966 patients treated with preoperative chemotherapy and radical cystectomy for invasive urinary bladder cancer. The martingale residual is the difference between the observed and the expected number of events for a given patient and demonstrates the expected risk of cancer-specific death for the patient. The figure suggest that patients’ age is not associated with the risk of cancer-specific death.

| **Supplementary table S1.** Univariable logistic regression investigating the association of age and age groups with pathologic objective response (pOR) and pathologic complete response (pCR) in 1,105 patients treated with neoadjuvant chemotherapy followed by radical cystectomy and lymphadenectomy | | | | | | |
| --- | --- | --- | --- | --- | --- | --- |
| Characteristic | Pathologic objective response | | | Pathologic complete response | | |
|  | OR*^1^* | 95% CI*^1^* | p-value | OR*^1^* | 95% CI*^1^* | p-value |
| Age (years) | 1.00 | 0.99, 1.01 | 0.7 | 1.00 | 0.99, 1.02 | 0.9 |
|  |  |  |  |  |  |  |
| Age groups |  |  |  |  |  |  |
| 27-57 | — | — |  | — | — |  |
| 58-64 | 1.13 | 0.81, 1.58 | 0.5 | 0.87 | 0.58, 1.29 | 0.5 |
| 65-71 | 0.92 | 0.65, 1.28 | 0.6 | 0.81 | 0.54, 1.21 | 0.3 |
| 72-87 | 0.88 | 0.61, 1.24 | 0.5 | 0.88 | 0.58, 1.33 | 0.6 |
| *^1^*OR = Odds Ratio, CI = Confidence Interval | | | | | | |

| **Supplementary table S2.** Survival analyses investigating the association of age with cancer specific survival (CSS), overall survival (OS) and death from bladder cancer in 377 patients with pathologic objective response after preoperative chemotherapy followed by radical cystectomy and lymphadenectomy | | | | | | | | | |
| --- | --- | --- | --- | --- | --- | --- | --- | --- | --- |
|  | Cox regression model (CSS) | | | Cox regression model (OS) | | | Fine and Gray model | | |
|  | HR | 95%CI | p | HR | 95%CI | P | HR | 95%CI | p |
| Age | 1.01 | 0.98, 1.04 | 0.8 | 1.01 | 0.98, 1.04 | 0.35 | 1.00 | 0.96, 1.04 | 0.88 |
| Age groups |  |  |  |  |  |  |  |  |  |
| 27-57 years | Ref. |  |  | Ref. |  |  | Ref. |  |  |
| 58-64 years | 0.81 | 0.39, 1.68 | 0.6 | 0.93 | 0.40, 2.20 | 0.9 | 0.96 | 0.41, 2.26 | 0.93 |
| 65-71 years | 0.56 | 0.24, 1.34 | 0.2 | 0.45 | 0.14, 1.44 | 0.2 | 0.46 | 0.14, 1.46 | 0.19 |
| 72-87 years | 1.37 | 0.65, 2.88 | 0.4 | 1.38 | 0.56, 3.39 | 0.5 | 1.38 | 0.57, 3.32 | 0.47 |
| HR = Hazard Radio, CI = Confidence Interval | | | | | | | | | |
